# Prevalence of Antimicrobial Resistance Genes in *Salmonella* Typhi: A Systematic Review and Meta-Analysis

**DOI:** 10.3390/tropicalmed7100271

**Published:** 2022-09-27

**Authors:** Nik Yusnoraini Yusof, Nur Iffah Izzati Norazzman, Nur Fatihah Mohd Zaidi, Mawaddah Mohd Azlan, Basyirah Ghazali, Mohamad Ahmad Najib, Abdul Hafiz Abdul Malik, Mohamad Aideil Helmy Abdul Halim, Muhammad Nor Syamim Mohd Sanusi, Annur Ashyqin Zainal, Ismail Aziah

**Affiliations:** 1 Institute for Research in Molecular Medicine (INFORMM), Health Campus, Universiti Sains Malaysia, Kubang Kerian 16150, Kelantan, Malaysia; 2 School of Health Sciences, Universiti Sains Malaysia, Kubang Kerian 16150, Kelantan, Malaysia

**Keywords:** prevalence, antimicrobial resistance gene, *Salmonella* Typhi, antibiotic resistance, systematic review, meta-analysis

## Abstract

*Salmonella* enterica serovar Typhi (*S.* Typhi) that has developed resistance to many antimicrobials poses a serious challenge to public health. Hence, this study aimed to systematically determine the prevalence of antimicrobial resistance (AMR) in *S.* Typhi isolated from the environment and humans as well as to ascertain the spread of the selected AMR genes in *S.* Typhi. This systematic review and meta-analysis were performed according to the Preferred Reporting Items for Systematic Review and Meta-Analysis (PRISMA) guidelines, and the study protocol was registered with the International Prospective Register of Systematic Reviews (PROSPERO). A total of 2353 studies were retrieved from three databases, of which 42 studies fulfilled the selection criteria. The pooled prevalence of AMR *S.* Typhi (using a random-effect model) was estimated at 84.8% (95% CI; 77.3–90.2), with high heterogeneity (*I*^2^: 95.35%, *p*-value < 0.001). The high estimated prevalence indicates that control methods should be improved immediately to prevent the spread of AMR among *S.* Typhi internationally.

## 1. Introduction

*Salmonella* enterica serovar Typhi (*S.* Typhi), a rod-shaped, gram-negative bacteria from the family Enterobacteriaceae, is responsible for typhoid fever, a serious bloodstream infection. It has been an important cause of morbidity and mortality in the developing world [1]. The incidence of typhoid in low- and middle-income countries in South Asia and Africa is more common than in developed countries [1]. The Global Burden of Disease Study estimated 9.25 million cases of typhoid fever in 2019, resulting in 110,000 deaths, of which the majority occurred in South Asia and sub-Saharan Africa [2]. In nature, *S.* Typhi can only infect humans and is usually contracted through ingestion of food or water contaminated with the organism’s excrement. It was reported that there were 888 cases and two deaths in Kelantan (Malaysia) during a large outbreak in 2005 [3]. Patients typically present symptoms after a 7-to-14 day asymptomatic period following ingestion of *S.* Typhi-contaminated food or water. Following the initial asymptomatic period, patients can develop an influenza-like illness with a fever and also nausea, vomiting and diarrhea [4].

It is well-known that antibiotics have reduced the morbidity and mortality of many infectious diseases [5] and have been used as an important weapon to fight typhoid fever. However, the spread and injudicious use of antibiotics in human medicine has led to the rapid emergence and spread of antimicrobial resistance (AMR) in *S.* Typhi strains [6]. There are various antibiotic-resistance-related genes found in *S.* Typhi including *catA1* (conferring resistance to chloramphenicol), *bla_TEM-1_* (resistant to ampicillin), *dhfr7*, and *sul1* (resistant to co-trimoxazole) [7]. The development of AMR in *S.* Typhi can occur spontaneously through mutations [8]. In addition, point mutations in the quinolone resistance-determining region (QRDR) harboring the genes for DNA gyrase *gyrA* and *gyrB* and topoisomerase IV *parC* and *parE* result in quinolone-resistant *S.* Typhi [7]. However, *S.* Typhi can also acquire the AMR gene from non-relatives on mobile genetic elements (MGE) such as plasmids and transposon. This horizontal gene transfer (HGT) allows the AMR gene to be transferred among different species of bacteria [9].

To date, the distribution of AMR in *S.* Typhi data among different countries, regions and environments is necessary for appropriate antimicrobial therapy for patient management and surveillance programs. Hence, in this study, a systematic review and meta-analysis were performed to evaluate the prevalence of AMR in *S.* Typhi isolated from the environment and humans globally as well as to assess the spread of the selected AMR genes in *S.* Typhi.

## 2. Materials and Methods

### 2.1. Study Design and Protocol

The present systematic review and meta-analysis were conducted following the preferred reporting items for systematic review and meta-analyses (PRISMA 2020) guidelines [10]. In addition, the protocol for this systematic review was registered in the International Prospective Register of Systematic Reviews (PROSPERO), with a registered ID of CRD42022319530.

### 2.2. Search Strategy

A systematic search was conducted on three databases, namely, PubMed, Scopus and Science Direct using the following keywords: (antimicrobial resistance gene) OR (antibiotic resistance gene) AND (*Salmonella* Typhi). To ensure comprehensive data collection, filters including publication year, study design and language were not applied in this review.

### 2.3. Inclusion and Exclusion Criteria

The studies’ titles and abstracts were screened based on the inclusion and exclusion criteria. Those articles that met the inclusion criteria were included and further reviewed. All of the eligible studies included in the review had to meet the following inclusion criteria: (i) *Salmonella* enterica serotype Typhi isolated from humans and environments, (ii) articles reported on antimicrobial resistance gene of *S.* Typhi and (iii) published in English. The studies were excluded if they did not involve the isolation of *S.* Typhi and non-resistant *S.* Typhi. Books, book chapters, review articles, media reports, case reports and studies written in languages other than English were also excluded from the review.

### 2.4. Data Extraction and Risk of Bias (RoB) Assessment

Studies that met the criteria for full-text review were selected for further analysis. Subsequently, risk assessment and data extraction were carried out. Selected studies were subjected to the following data extraction: (i) first author; (ii) period of study; (iii) region and country of the conducted studies; (iv) methodology: the total number of samples, type of samples (patient/clinical, environment), and the total number of *S.* Typhi isolated and; (v) outcome: number of isolates resistance against each antimicrobial, number of bacteria isolates with AMR gene and number of mutated cases. In addition, data on the type of antibiotics resistant among *S.* Typhi was also extracted. Studies that collected samples from different countries and regions were categorized as worldwide in order to avoid confusion during data extraction and analysis.

The Joanna Briggs Institute (JBI) critical appraisal tool for Studies Reporting Prevalence Data was used to assess the risk of bias (RoB) of the selected studies. Parameters assessed for bias included (i) sample description, i.e., either from an environmental or hospital setting; (ii) study design, sample size and sampling techniques; (iii) use of valid and standard methods in microbiologic techniques such as bacteriologic culture and antimicrobial sensitivity; (iv) confirmation methods for detection of AMR genes in *S.* Typhi and (v) the statistical analysis used for reporting summary measures. The quality of each study was graded with ‘1’ for an answer to each question of ‘yes’ and ‘0’ for answers of ‘no’ or ‘unclear’. The low-RoB articles (the total quality score was ≥7) were included in the study whereas articles with moderate (the total quality score was between 4 and 6) and high RoB (the total quality score was ≤3) were excluded for further analysis.

### 2.5. Data Synthesis and Analysis

Data analysis was performed using Comprehensive Meta-Analysis Software (CMA) (Version 3.0). The pooled prevalence of AMR in *S.* Typhi, and the selected AMR genes (*gyrA*, *gyrB*, *parC*, *parE* and *bla_TEM_* genes) in *S.* Typhi were measured and the sub-group analysis (to analyze the sources of heterogeneity) was conducted according to the country and region of each study. A random-effect model using the DerSimonian-Laird method of meta-analysis was employed to create the pooled estimates of the reported AMR among *S.* Typhi and the chosen AMR genes in *S.* Typhi cases. A forest plot was generated to summarize details of the individual studies alongside the estimated common effect and degree of heterogeneity. The potential for publication bias was examined using funnel plots (visual aid for detecting bias) and the asymmetry of the plot was further assessed using Egger’s regression test. The heterogeneity (i.e., variation in study outcomes between studies) of the study-level estimates was evaluated using Cochran’s Q test and *I*^2^ estimates. *I*^2^ values of 25%, 50% and 75% were considered low, moderate and high heterogeneity, respectively [11]. For all of the tests, a *p*-value of <0.05 was considered to be statistically significant.

## 3. Results

### 3.1. Overview of the Selected Studies

**Figure 1:**
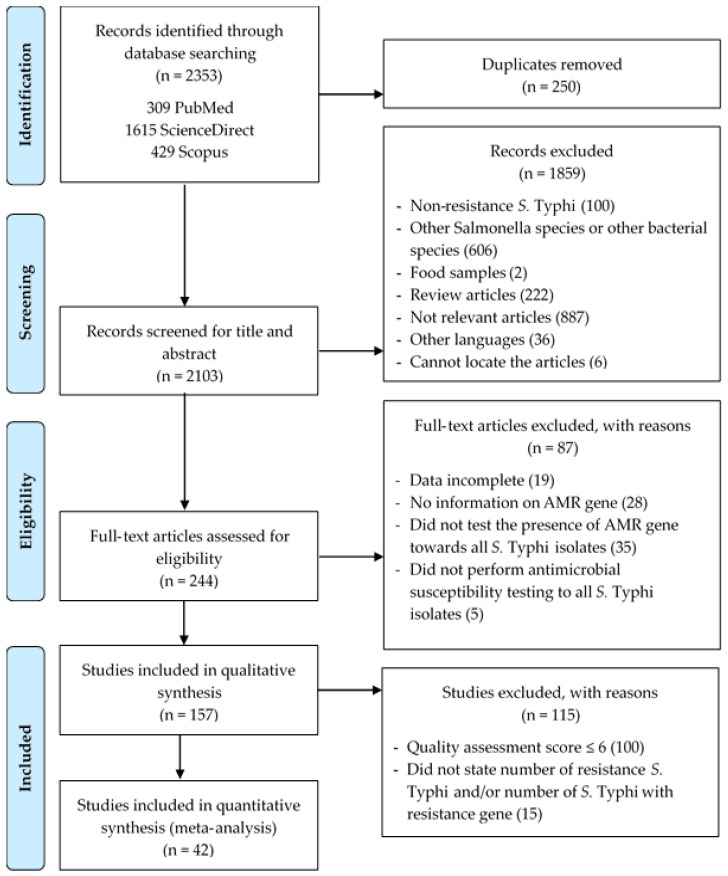
PRISMA flowchart illustrating the process of identifying, screening and selecting eligible articles used in this study.

A total of 2353 studies were retrieved from the targeted online databases, of which 250 duplicates were removed, as presented in the PRISMA flowchart (Figure 1). The titles and abstracts of 2103 studies were screened based on the inclusion and exclusion criteria and 1859 studies were excluded for further analysis. As a result, 244 full texts were assessed for eligibility. A total of 87 studies were removed due to incomplete data on the number of *S.* Typhi isolates, AMR genes, tests used for the detection of the AMR genes and antimicrobial susceptibility tests for all *S.* Typhi. A total of 157 studies were included in the final qualitative synthesis before removing 115 studies due to the high and moderate risk of bias based on the risk quality assessment score (≤6 score), a lack of detail on the number of resistant *S.* Typhi and/or the number of *S.* Typhi with resistance gene being unspecified. Finally, 42 studies were included in the meta-analysis.

### 3.2. Characteristics of the Eligible Studies

All the eligible studies included in the meta-analyses were of high methodological quality (Appendix A; Table A1). Most of the included studies were conducted in Asia (n = 23) involving nine countries, where India is the main country of origin of the isolates reported in eligible studies (n = 7) (Table 1). Isolates were also collected from Bangladesh and Pakistan; both countries provided four studies. Samples originating from China constituted three studies (n = 3) and the remaining were from Cambodia, Indonesia, Japan, Myanmar and Nepal; each constituted one study from the eligible articles (n = 1).

**Figure 2:**
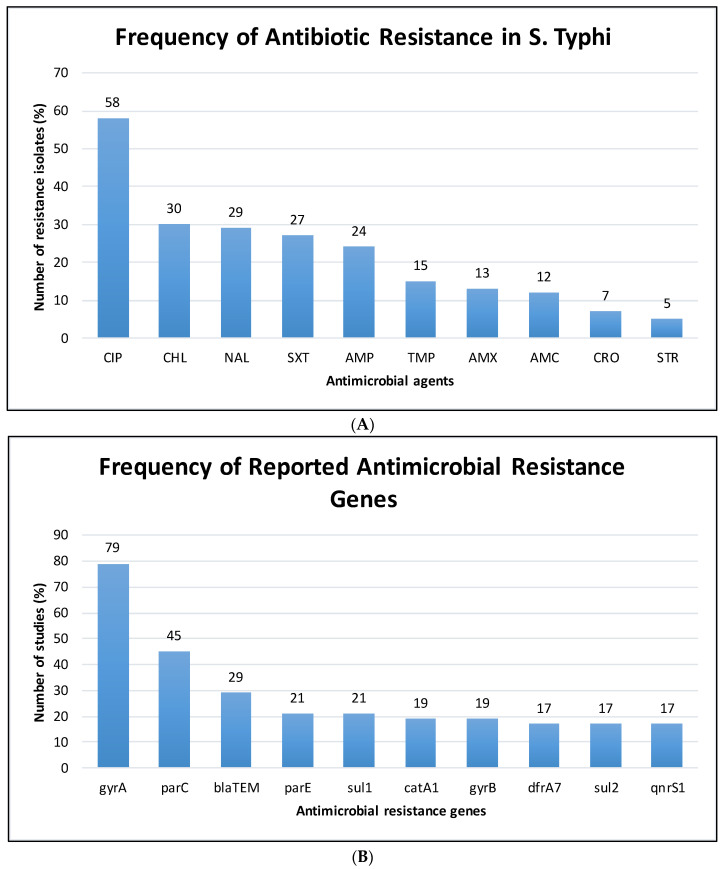
The frequency of top ten (**A**) antibiotic resistance in *S.* Typhi and (**B**) reported antimicrobial resistance genes. AMC: amoxicillin-clavulanic acid, AMP: ampicillin, AMX: amoxicillin, CHL: chloramphenicol, CIP: Ciprofloxacin, NAL: nalidixic acid, CRO: ceftriaxone, STR: streptomycin, SXT: trimethoprim-sulfamethoxazole and TMP: trimethoprim.

A total of 4359 isolates derived from humans (patient/clinical) and environmental sources were tested for phenotypic and genotypic antimicrobial resistance profiles. Of all the selected studies, the highest proportions of *S.* Typhi isolates were identified from human samples. Of the 42 studies, 39 confirmed that 4242 samples of *S.* Typhi were isolated from humans (Table 1). Meanwhile, 113 strains were isolated from the environment, as reported by two included studies [16,44]. However, it was unclear from one study how many of the four *S.* Typhi isolates originated in humans versus the environment [27]. Among the isolates, 3320 were resistant to at least one antimicrobial agent. The majority of the isolates were resistant to ciprofloxacin (58%), chloramphenicol (30%) and nalidixic acid (29%) (Figure 2A). Only one study by Al-Muhanna et al. [21] did not reveal the phenotypic antibiotic resistance profile as the report highlighted only the number of resistant *S.* Typhi isolates (Table 1). Our analysis showed a total of 42 genes conferring antibiotic resistance. The most reported AMR genes were the *gyrA* (79%) and *parC* (45%) genes, both genes conferring resistance quinolones as well as the *bla_TEM_* gene (29%), conferring resistance against β-lactam (Figure 2B).

Apart from that, the distribution of mutations in *gyrA*, *gyrB*, *parC* and *parE* genes was also retrieved from the eligible articles (Appendix B; Table A2). Out of 34 studies that reported the presence of the *gyrA* gene in resistant *S.* Typhi, 30 studies showed mutations in *gyrA* while the remaining studies did not test for the presence of *gyrA* mutations in the isolates. Mutations at codon 83 (S83F) and 87 (D87N) were the most commonly reported mutations in the *gyrA* gene (S83F: n = 28 studies, D87N: n = 16 studies). Mutations in the *gyrB* gene were reported in seven studies in which S464F (n = 4 studies) and S464Y (n = 2 studies) mutations were the most frequently reported. The most commonly reported mutations in topoisomerase IV genes (*parC* and *parE*) were S80I (*parC*) (n = 15 studies) and D420N (*parE*) (n = 3 studies).

### 3.3. The Pooled Prevalence of Antimicrobial Resistant Strains in S. Typhi

**Figure 3:**
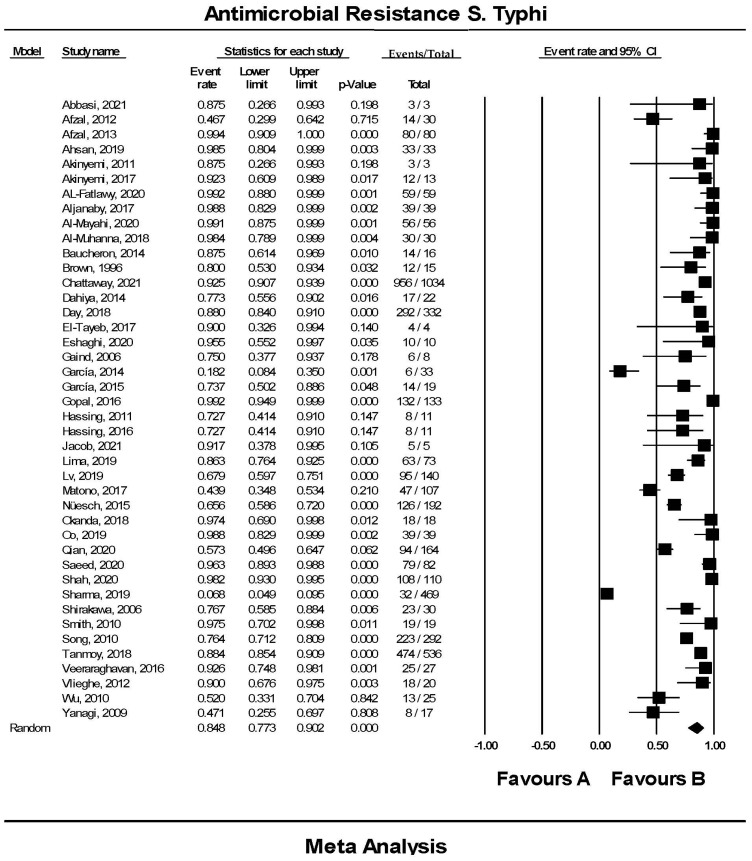
Forest plot showing the pooled prevalence of resistant *S.* Typhi isolates estimated by a random effect model of meta-analysis (84.8%, *I*^2^: 95.35, 95% CI: 77.3–90.2, *p*-value < 0.001) [12,13,14,15,16,17,18,19,20,21,22,23,24,25,26,27,28,29,30,31,32,33,34,35,36,37,38,39,40,41,42,43,44,45,46,47,48,49,50,51,52,53].

**Figure 4:**
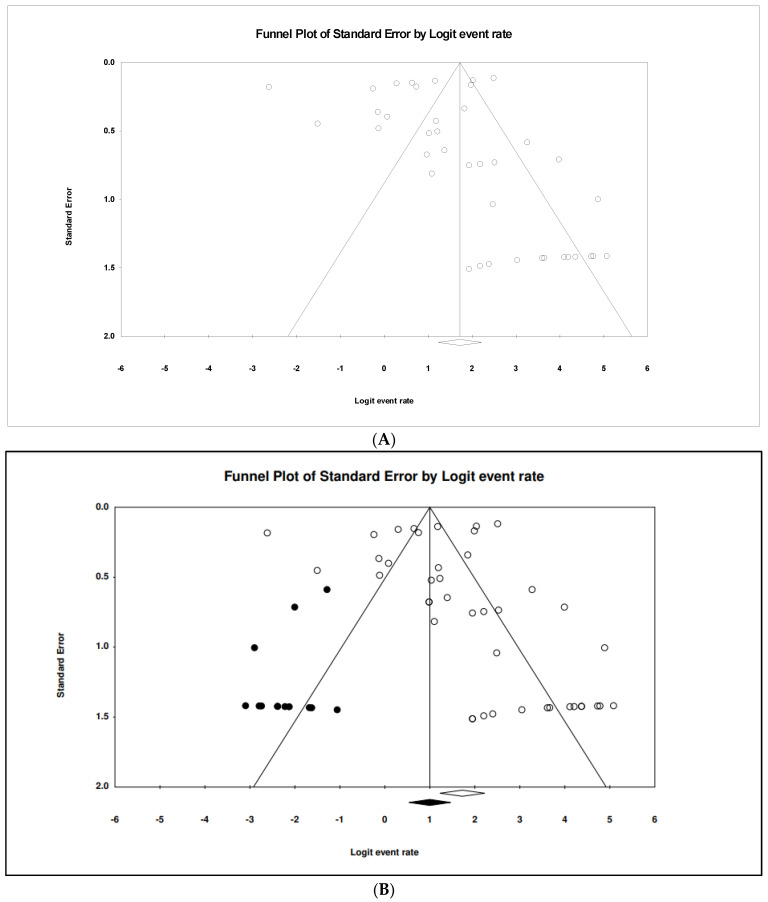
Funnel plot showing (**A**) publication bias in studies reporting the prevalence of antimicrobial resistant *S.* Typhi and (**B**) result after performing the trim-and-fill method where 13 missing studies (closed circles) were added on the left side of the mean effect.

The pooled prevalence of AMR strains in *S.* Typhi using a random-effect model was estimated at 84.8% (95% CI; 77.3–90.2), but with high heterogeneity (*I*^2^ = 95.35%, *p*-value < 0.001) (Figure 3). A sub-group analysis based on countries and regions was performed to examine the potential sources of heterogeneity, which were presented in Table 2. The highest pooled prevalence of 98.9% (95% CI; 95.7–99.7) was observed from Iraq (n = 4) and the lowest pooled prevalence was estimated at 18.2% (95% CI, 8.4–35.0) from Peru (n = 1) Heterogeneity was highest among studies conducted in India (*I*^2^ = 96.529%) followed by four studies conducted in Pakistan (*I*^2^ = 93.686%). Based on regional data, studies from America (n = 2) showed the lowest estimate at 63.7% (95% CI; 2.0–99.3), had *I*^2^ of 88.889%, and studies from the Middle East region (n = 6) showed the highest estimate at 97.7% (95% CI; 93.0–99.3) with *I*^2^ of 0.000%. In addition, the funnel plot showed evidence of publication bias attributed to the relatively asymmetrical plot (Figure 4A). Moreover, using the trim-and-fill method (under the random effects model), 13 missing studies were imputed to the left side of the mean effect, resulting in the imputed point estimate of 73.080% (Figure 4B). In addition to the funnel plots, Egger’s test was utilized to affirm the extent of bias (*t*-value = 0.938, *p*-value = 0.177).

### 3.4. The Pooled Prevalence of gyrA Gene in S. Typhi

**Figure 5:**
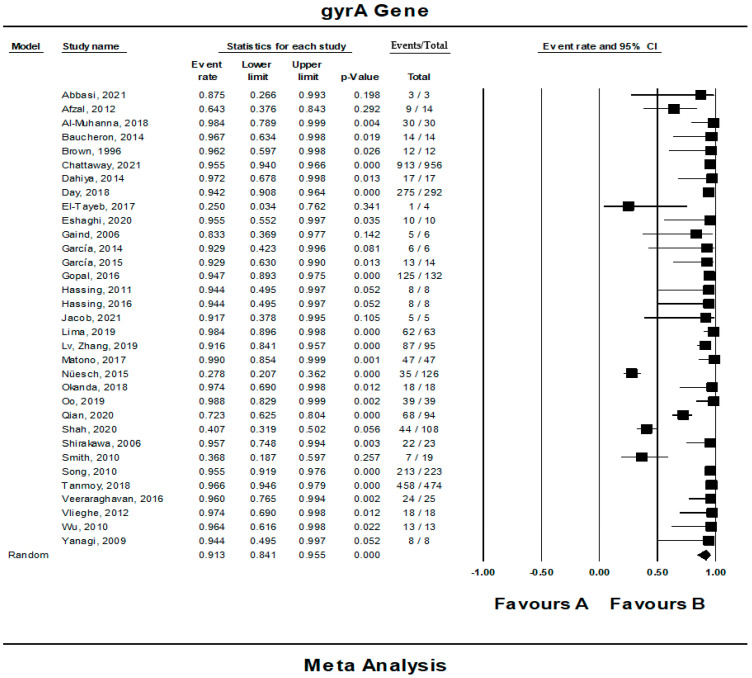
Forest plot showing the pooled prevalence of *gyrA* gene in resistant *S.* Typhi isolates estimated by a random effect model of meta-analysis. (91.3%, *I*^2^: 93.99, 95% CI: 84.1–95.5, *p*-value < 0.001) [12,13,14,15,16,17,18,19,20,21,22,23,24,25,26,27,28,29,30,31,32,33,34,35,36,37,38,39,40,41,42,43,44,45,46,47,48,49,50,51,52,53].

**Figure 6:**
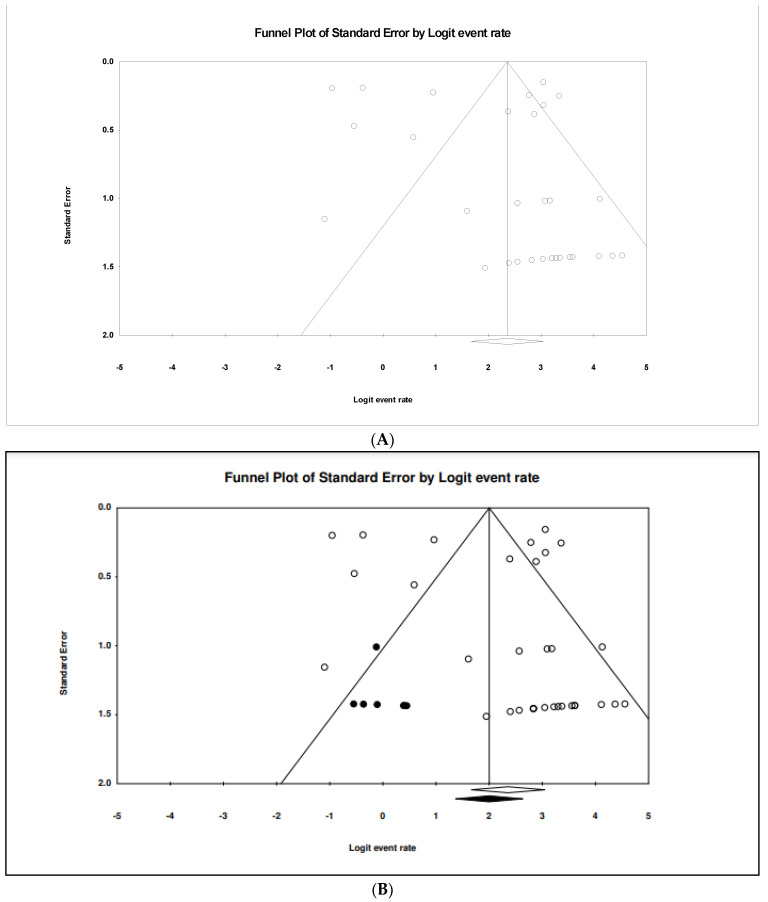
Funnel plot showing (**A**) publication bias in studies reporting the prevalence of *gyrA* gene in antimicrobial resistant *S.* Typhi and (**B**) result after performing the trim-and-fill method where seven missing studies (closed circles) were added on the left side of the mean effect.

Out of 42 included studies, only 33 studies reported the presence of the *gyrA* gene in *S.* Typhi isolates. The prevalence and heterogeneity of the *gyrA* gene in resistant *S.* Typhi are presented in Figure 5. Of the 2922 isolates, the prevalence of *gyrA* resistance was 91.3% (95% CI; 84.1–95.5), indicating that the majority of the resistant *S.* Typhi possessed the *gyrA* gene. The heterogeneity of the included studies was significantly high (*I*^2^ = 93.99%, *p*-value < 0.001). The pooled prevalence based on country revealed Japan with one study as the highest (99.0%, 95% CI; 845.4–99.9) with a heterogeneity of 0.000 (Table 3). The lowest prevalence was observed in Saudi Arabia (n = 1), 25.0% (95% CI; 3.4–76.2) with a heterogeneity of 0.000. Sub-group analysis based on regions showed that America (n = 2) had the highest estimation, 94.3% (*I*^2^ = 0.000%, 95% CI; 68.7–99.2), followed by Asia (n = 19), 93.2% (*I*^2^ = 91.108%, 95% CI; 85.2–97.0). However, the asymmetrical funnel plot showed the presence of publication bias (Figure 6A). The trim-and-fill method (under the random effects model) showed a point estimate of 88.092% with seven missing studies imputed to the left side of the mean effect (Figure 6B). In addition to the funnel plots, Egger’s test was utilized to affirm the extent of bias (*t*-value = 1.158, *p*-value = 0.128).

### 3.5. The Pooled Prevalence of gyrB Gene in S. Typhi

**Figure 7:**
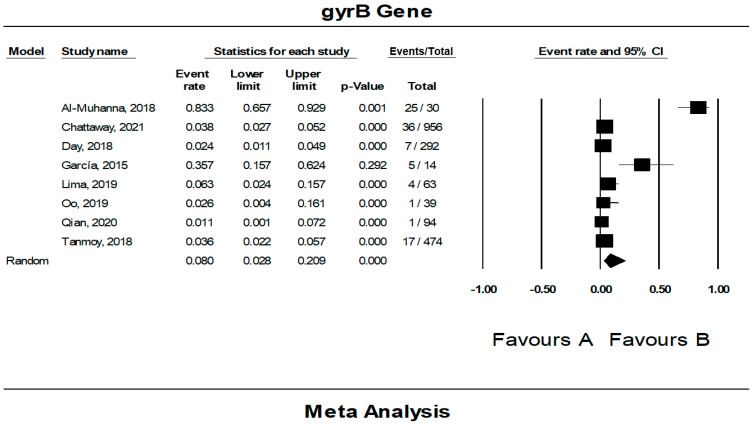
Forest plot showing the pooled prevalence of *gyrB* gene in resistant *S.* Typhi isolates estimated by a random effect model of meta-analysis. (8.0%, *I*^2^: 93.98, 95% CI: 2.8–20.9, *p*-value < 0.001) [21,24,26,31,36,41,42,49].

**Figure 8:**
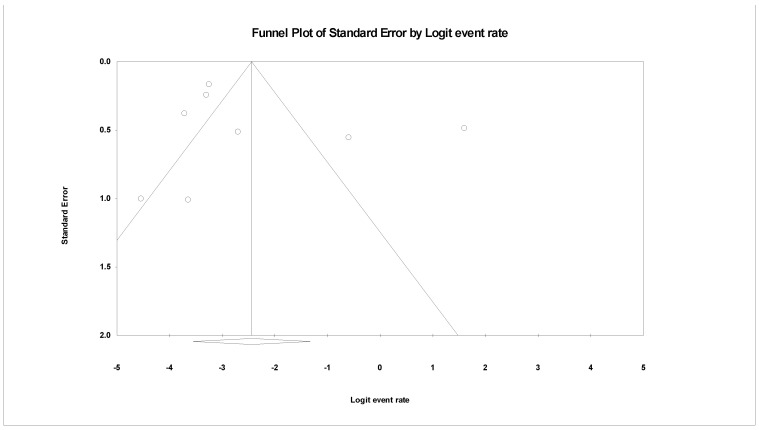
Funnel plot showing publication bias in studies reporting the prevalence of *gyrB* gene in antimicrobial resistant *S.* Typhi.

Eight of the 42 publications showed data for the *gyrB* gene in resistant *S.* Typhi. Figure 7 presents a forest plot of the proportions of *gyrB* gene in resistant isolates. The pooled prevalence was estimated at 8.0% out of 1962 resistant isolates indicating that the *gyrB* gene was quite rare in these isolates. Overall, data heterogeneity was similar in the *gyrA* gene (*I*^2^ = 93.98%, *p*-value < 0.001). On the other hand, the pooled prevalence based on country showed Iraq, with one study, as the highest (83.3%, 95% CI; 65.7–92.9) with a heterogeneity of 0.000% (Table 4). The lowest prevalence was observed in the United Kingdom (n = 1), 2.4% (95% CI; 1.1–4.9) with a heterogeneity of 0.000%. By region, the Middle East (n = 1) had the highest estimation, 83.3% (*I*^2^ = 0.000%, 95% CI; 65.7–92.9), followed by Europe (n = 3), 6.9% (*I*^2^ = 91.477%, 95% CI; 1.9–22.5). In addition, the asymmetrical funnel plot showed the presence of publication bias (Figure 8). However, further tests on this asymmetrical funnel plot could not be performed as the number of studies included for the analysis of the *gyrB* gene was less than 10 (n = 8) [54].

### 3.6. The Pooled Prevalence of parC Gene in S. Typhi

**Figure 9:**
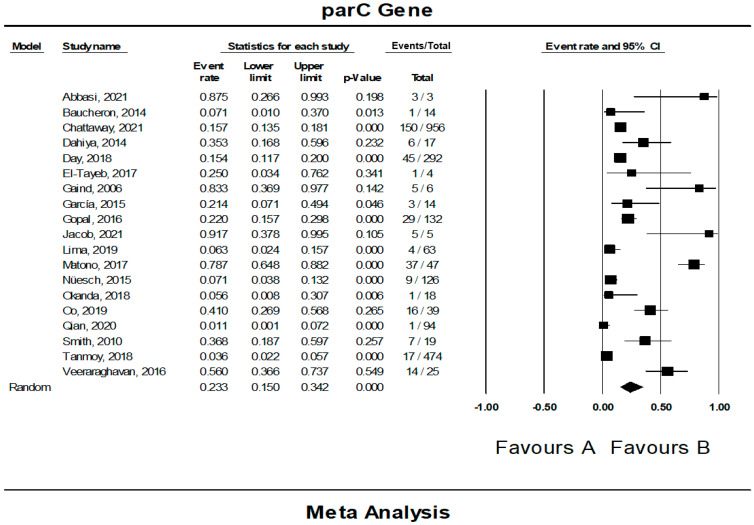
Forest plot showing the pooled prevalence of *parC* gene in resistant *S.* Typhi isolates estimated by a random effect model of meta-analysis. (23.3%, *I*^2^: 91.26, 95% CI: 15.0–34.2, *p*-value < 0.001) [12,22,24,25,27,29,31,32,35,36,38,39,40,41,42,47,49,50].

**Figure 10:**
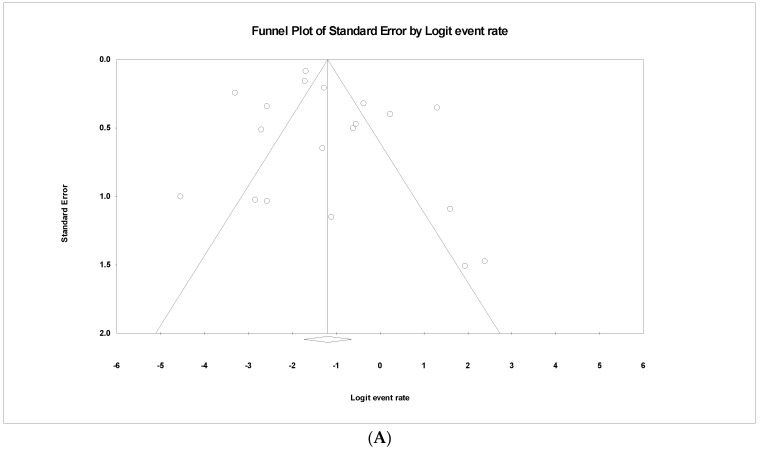

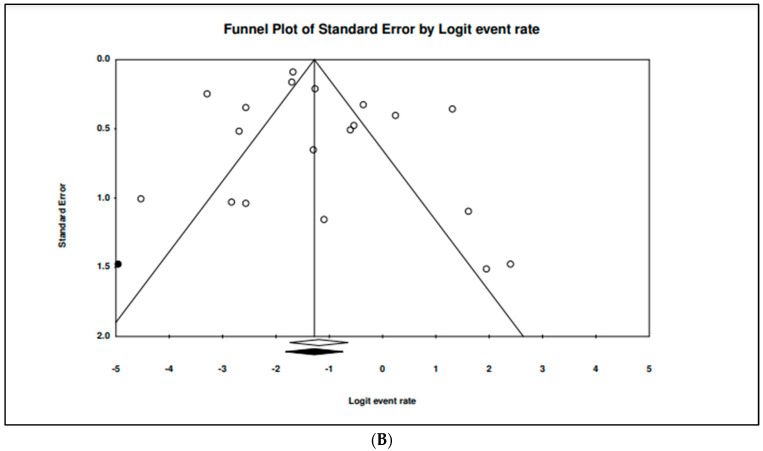
Funnel plot showing (**A**) publication bias in studies reporting the prevalence of *parC* gene in antimicrobial resistant *S.* Typhi and (**B**) result after performing trim-and-fill method where one missing study (closed circles) were added on the left side of the mean effect.

Out of 42 included studies, only 19 studies reported the presence of the *parC* gene in resistant *S.* Typhi isolates. The prevalence and heterogeneity of the *parC* gene in resistant *S.* Typhi are presented in Figure 9. Of the 2348 isolates, the prevalence of the *parC* gene resistance was 23.3% (95% CI; 15.0–34.2), indicating that the *parC* gene was less dominant in the resistant *S.* Typhi. Heterogeneity analysis showed that data heterogeneity was significantly high (*I*^2^ = 91.26%, *p*-value < 0.001). Sub-group analysis by country revealed that the prevalence of *parC* gene resistant was highest in Iran (n = 1) at 87.5% (95% CI; 26.6–99.3) with heterogeneity of 0.000% (Table 5). The lowest prevalence was observed in China (n = 1), 1.1% (95% CI; 0.1–7.2) with heterogeneity of 0.000%. Interestingly, France and Switzerland, with the same number of studies (n = 1) showed a similar prevalence of 7.1% (95% CI; 1.0–37.0 (France), 3.8–13.2 (Switzerland)) and heterogeneity of 0.000%. Based on regions, the Middle East (n = 2) had the highest prevalence at 56.6% (95% CI; 6.3–96.2) compared to other regions. Meanwhile, the lowest prevalence was reported in Europe (n = 5), 14.0% (95% CI; 10.8–18.0). Additionally, the asymmetrical funnel plot showed the presence of publication bias (Figure 10A). Moreover, the trim-and-fill method (under the random effects model) showed a point estimate of 21.750% with one missing study imputed to the left side of the mean effect (Figure 10B). In addition to the funnel plots, Egger’s test was utilized to affirm the extent of bias (*t*-value = 1.085, *p*-value = 0.147).

### 3.7. The Pooled Prevalence of parE Gene in S. Typhi

**Figure 11:**
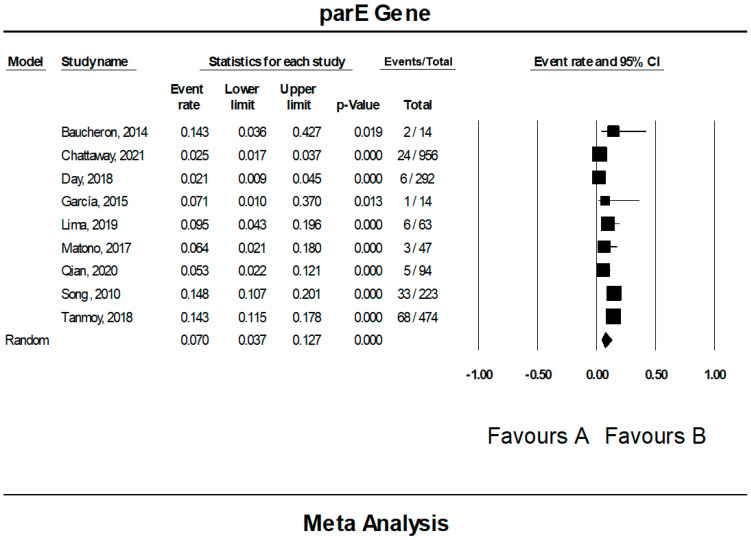
Forest plot showing the pooled prevalence of *parE* gene in resistant *S.* Typhi isolates estimated by a random effect model of meta-analysis. (7.0%, *I*^2^: 90.47, 95% CI: 3.7–12.7, *p*-value < 0.001) [22,24,26,31,36,38,42,48,49].

**Figure 12:**
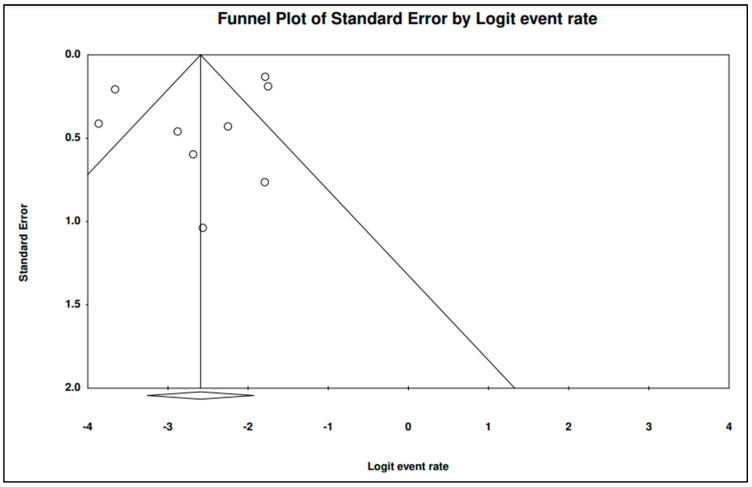
Funnel plot showing publication bias in studies reporting the prevalence of *parE* gene in antimicrobial resistance *S.* Typhi.

Nine of the 42 publications showed data for the *parE* gene in resistant *S.* Typhi. Figure 11 presents a forest plot of the proportions of the *parE* gene in resistant isolates. The pooled prevalence was estimated at 7.0% out of 2177 resistant isolates, indicating that the *parE* gene was quite rare in these isolates. Overall, the heterogeneity in data across the eligible studies was significantly high (*I*^2^ = 90.47%, *p*-value < 0.001). On the other hand, the pooled prevalence by country showed Worldwide with one study as the highest (14.8%, 95% CI; 10.7–20.1) with a heterogeneity of 0.000% (Table 6). The lowest prevalence was observed in the United Kingdom (n = 1), 2.1% (95% CI; 0.9–4.5) with a heterogeneity of 0.000%. By region, the highest estimate was also observed as Worldwide (n = 1) 14.8% (95% CI; 10.7–20.1, *I*^2^ = 0.000%,), while the lowest prevalence was observed Europe (n = 4) 3.6% (95% CI; 1.7–7.2, *I*^2^ = 57.466%,). In addition, the asymmetrical funnel plot showed presence of publication bias (Figure 12). However, further tests on this asymmetrical funnel plot could not be performed as the number of studies included for the analysis of *parE* gene was less than 10 (n = 9) [54].

### 3.8. The Pooled Prevalence of bla_TEM_ Gene in S. Typhi

**Figure 13:**
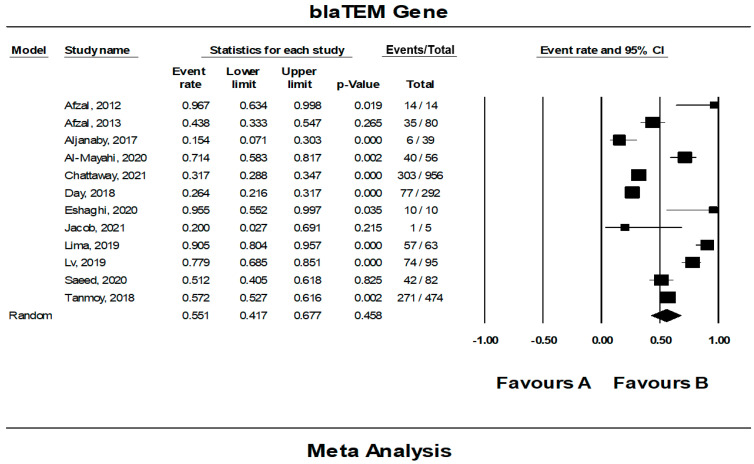
Forest plot showing the pooled prevalence of *bla_TEM_* gene in resistant *S.* Typhi isolates estimated by a random-effect model of meta-analysis. (55.1%, *I*^2^: 95.27, 95% CI: 41.7–67.7, *p*-value < 0.500) [13,14,19,20,24,26,28,35,36,37,43,49].

**Figure 14:**
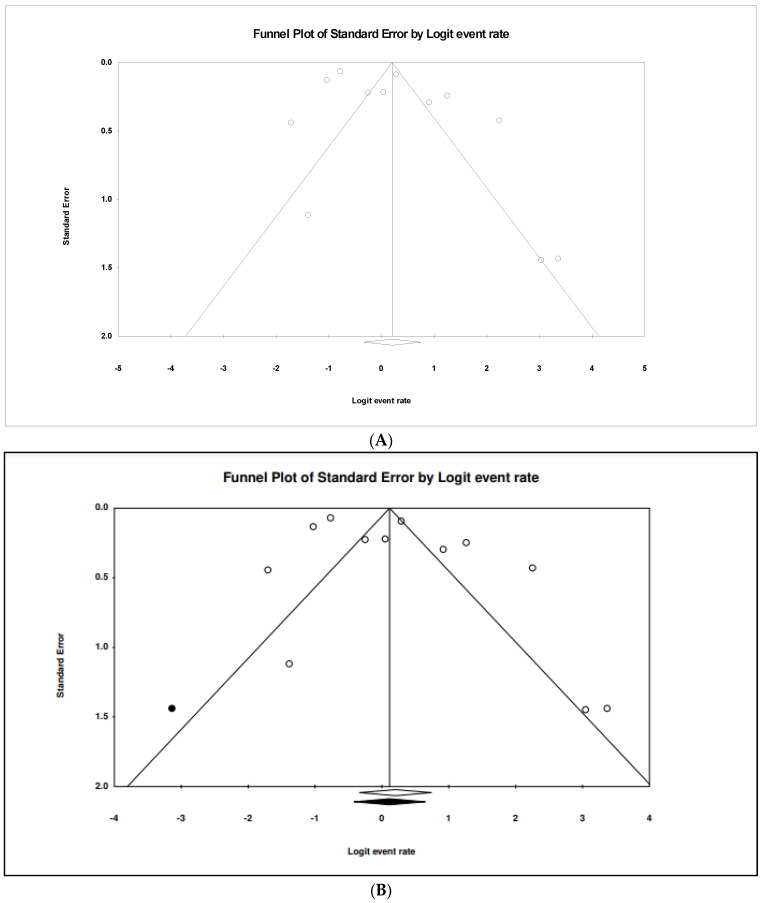
Funnel plot showing (**A**) publication bias in studies reporting the prevalence of *bla_TEM_* gene in antimicrobial resistant *S.* Typhi and (**B**) result after performing trim-and-fill method where one missing study (closed circles) was added on the left side of the mean effect.

Out of 42 included studies, only 12 studies reported the presence of the *bla_TEM_* gene in resistant *S.* Typhi isolates. The prevalence and heterogeneity of the *bla_TEM_* gene in resistant *S.* Typhi are presented in Figure 13. Of the 2166 isolates, the prevalence of *bla_TEM_* in resistant isolates was 55.1% (95% CI; 41.7–67.7), indicating that the majority of the resistant *S.* Typhi possessed the *bla_TEM_* gene. The heterogeneity in the data was high but at an insignificant level (*I*^2^ = 95.27, *p*-value < 0.500). The sub-group analysis based on countries revealed the highest estimate of 95.5% in Canada (n = 1) (95% CI; 55.2–99.7) and had low heterogeneity, 0.000% (Table 7). The lowest prevalence was observed in India (n = 1), 20.0% (95% CI; 2.7–69.1) with a heterogeneity of 0.000%. In terms of regions, America (n = 1) had the highest prevalence at 95.5% (95% CI; 55.2–99.7) compared to other regions. The lowest prevalence was reported in Europe (n = 2), 29.5% (95% CI; 24.6–34.9). Additionally, the asymmetrical funnel plot showed the presence of publication bias (Figure 14A). Moreover, trim-and-fill analysis (under the random effects model) showed a point estimate of 52.870% with one missing study imputed to the left side of the mean effect (Figure 14B). In addition to the funnel plots, Egger’s test was utilized to affirm the extent of bias (*t*-value = 1.463, *p*-value = 0.087).

## 4. Discussion

*S.* Typhi infection has recently grown to be a serious burden to healthcare systems, causing higher morbidity and mortality among patients worldwide. Effective choices for the treatment of *S.* Typhi infections are becoming challenging as resistant strains are increasing globally [5]. Therefore, in the present review, a meta-analysis was performed to estimate the global prevalence of antimicrobial resistance in *S.* Typhi isolated from the environment and humans. A total of 42 studies that met the inclusion criteria were included for final analysis. Of these, there were 3320 AMR *S.* Typhi isolates recorded from 23 countries in six regions. A random-effects model was used to analyze the data. As a result, the pooled prevalence of AMR in *S.* Typhi was estimated to be 84.8% (95% CI; 77.3–90.2).

In general, the prevalence of AMR was high in isolates from the Middle East (97.7%), Africa (93.5%) and Asia (83.5%). These regions are known to be endemic areas for enteric fever where good sanitation and better public health management are not widely practiced [1]. The higher prevalence of AMR was due to the indiscriminate use of antibiotics [52]. Owing to high living expenses, patients in low- and middle-income countries may not have access to or be able to visit health institutions, forcing them to seek care elsewhere, such as in the informal sector or over the counter at local pharmacies [55]. Across our investigation, a significant amount of AMR in Europe (81.4%) was also reported. However, travelers returning from endemic regions and guests visiting family and friends, who are more inclined to be negligent with food and water sources, accounted for the majority of cases [1].

Our analysis found that heterogeneity between studies was high (*I*^2^ = 95.35%, *p*-value < 0.001), contributed by India (*I*^2^ = 96.529%) and Pakistan (*I*^2^ = 93.686%). The remaining countries were low heterogeneity (*I*^2^ = 0.000–22.168%), except for China which had moderate heterogeneity (*I*^2^ = 55.694%). In terms of regions, the higher heterogeneity was contributed by Asia (*I*^2^ = 96.156%), Europe (*I*^2^ = 93.997%) and America (*I*^2^ = 88.889%). The other regions, Africa (*I*^2^ = 0.000%), Middle East (*I*^2^ = 0.000%) and worldwide (*I*^2^ = 0.000%), showed a low heterogeneity. The different methods and interpretations of “resistant” *S.* Typhi may contribute to the high heterogeneity, especially in Asia and Europe. For instance, Afzal et al. [13] used Kirby–Bauer disk diffusion and E-test strips methods and interpreted the results using Clinical and Laboratory Standards Institution (CLSI) 2012 guidelines. In contrast, Lima et al. [36] used disk diffusion and broth-microdilution methods and interpreted the results according to the European Committee on Antimicrobial Susceptibility Testing (EUCAST v8) 2018. Our results show that Asia persists as a major source of antimicrobial resistance in *S.* Typhi and that clones of the bacteria that develop resistance in Asia are frequently spread throughout the region and beyond. These findings demonstrate the widespread dissemination of antimicrobial-resistant *S.* Typhi strains internationally, emphasizing the need to consider typhoid management strategies from a global rather than a regional perspective. Antimicrobials have contributed to controlling and eliminating infectious diseases. However, the improper use of these drugs has caused the emergence of resistance strains, and, in turn, very limited drugs are left to treat the disease. Thus, the discovery of new drugs within a particular class of antimicrobials is required to replace existing drugs that have been ineffective. Furthermore, the improvement in antibiotic stewardship plays a significant role in the maintenance of the resistance phenotype and could have a beneficial impact on the proper management of antimicrobial resistant strain outbreaks.

A total of 42 genes conferring antibiotics resistance against *S.* Typhi isolates were observed. However, only five genes which include *gyrA*, *gyrB*, *parC*, *parE* and *bla_TEM_* were chosen to evaluate their prevalence. These genes were chosen based on the highest frequency of the top five genes that have contributed to the continuous emergence of quinolone- and β-lactam-resistant isolates in *S.* Typhi [56]. Both quinolones and β-lactam were commonly prescribed to treat *S.* Typhi-infected individuals [55]. However, the emergence of quinolones- and β-lactam-resistant isolates due to these resistance genes may cause a challenge in the treatment of *S.* Typhi infection and regulatory safeguards are required for the use of these antibiotic classes. The *gyrA*, *gyrB*, *parC* and *parE* genes were associated with resistant to quinolones [57]. The *gyrA* and *gyrB* genes encode for the DNA gyrase subunit while *parC* and *parE* genes encode for the topoisomerase IV subunit [58]. These enzymes are the target sites of quinolones to inhibit DNA replication and cause cell death in the bacteria [57]. However, mutations in these genes, overexpression of the efflux pump system and the innate impermeability of the membrane result in quinolone resistance [59]. On the other hand, the *bla_TEM_* gene was associated with resistance to β-lactam [60]. It encodes for β-lactamases that cause enzymatic inactivation (e.g., acetylation of aminoglycosides) and degradation of the β-lactam [61,62].

Based on the result of the analysis, a high prevalence was reported in the *gyrA* gene, 91.3%, followed by the *bla_TEM_* gene, 55.1%, and the *parC* gene, 23.3%. In contrast, both *gyrB* and *parE* genes were relatively rare in the resistant isolates in which the prevalence of these genes was 8.0% and 7.0% respectively. Based on sub-group analysis, America showed high prevalence in *gyrA* (94.3%, 95% CI; 68.7–99.2) and *bla_TEM_* genes (95.5%, 95% CI; 55.2–99.7), while the Middle East showed high prevalence in *gyrB* (83.3%, 95% CI; 65.7–92.9) and *parC* genes (56.6%, 95% CI; 6.3–96.2). On the other hand, the *parE* gene was highly reported worldwide (14.8%, 95% CI; 10.7–20.1). The pooled prevalence of the *gyrA* gene in Africa reported by Tadesse et al. [63] was 5.7% which was found to be slightly lower compared to our study (Africa = 36.8%, 95%; 18.7–59.7).

Mutations in quinolone-resistance genes were also observed in which mutations in *gyrA* and *parC* genes were the most commonly reported. According to Nguyen Van et al. [63] there is a strong association between changes in the *gyrA* and *parC* genes and the minimum inhibitory concentrations (MICs) of the antibiotics levofloxacin (LEV) and ciprofloxacin (CIP). The *gyrA* and *parC*’s quinolone resistance-determining region (QRDR) is where most mutations that cause fluoroquinolone resistance can be identified [64]. In our study, the most common *gyrA* mutations occurred in codons Ser-83 (n = 29 studies) and Asp-87 (n = 21 studies). On the other hand, the most common *parC* mutations occurred in codons Ser-80 (n = 15 studies) and Glu-84 (n = 10 studies). These data were consistent with the findings reported by Fukushima et al. [65] in which most mutations in the QRDR are at *gyrA* Ser-83 and Asp-87 and at *parC* Ser-80 and Glu-84.

Despite the comprehensive review, there are few limitations in this study. First, the meta-analysis could not cover all of the countries and regions in order to understand the complete overview of prevalence in AMR *S.* Typhi and the AMR gene in AMR *S.* Typhi, which is attributed to the lack of data in some countries. Second, since this review excluded studies written in language other than English, we might have omitted data from some countries, resulting in a publication language bias. In addition, most of the isolates were derived from typhoid cases, and the numbers of isolates from the environmental samples were relatively limited. In summary, more surveillance data are required to adequately reflect the resistant isolates from various countries to overcome the limitations. Moreover, this study was unable to cover all antimicrobial resistance genes to provide a comprehensive view of the prevalence of antimicrobial resistance genes as some of the data on these genes were limited. Other than that, in order to achieve high accuracy of the overall prevalence of antimicrobial resistance genes, studies that did not report the study period are also included in this review as these studies provide data on the prevalence of antimicrobial resistance genes in *S.* Typhi. Despite this caveat, findings of the present review may still provide useful insights for government decision makers and health practitioners on the urgency to pay attention towards preparing for dealing with the emergence of *S.* Typhi antibiotic resistance.

## 5. Conclusions

In conclusion, this systematic review and meta-analysis provided an overview of AMR *S.* Typhi in humans and the environment. The pooled prevalence of AMR *S.* Typhi was estimated at 84.8% (95% CI; 77.3–90.2), which was considerably high. Based on the 42 studies, most *S.* Typhi isolates were resistant to ciprofloxacin, chloramphenicol, nalidixic acid and ampicillin. Mutations in *gyrA* and *parC* genes were the most commonly reported whereas mutation in *gyrB* and *parE* was rarely reported. These mutations may contribute to the resistance of quinolone in *S.* Typhi. The establishment of AMR *S.* Typhi and the propagation of AMR-related genes data must be regularly monitored to provide information needed for management decisions about the use of antibiotics. Preventive actions should be taken immediately to prevent the spread of AMR *S.* Typhi internationally, and there is a need for a collaborative study on the epidemiology of AMR development and essential intervention across the human health sectors, as well as environmental sector.

We conclude that with the emergence of typhoid infection by resistant *S.* Typhi, surveillance programs including health awareness should be carried out effectively to reduce cases and control the disease.

## Figures and Tables

**Table 1:** Major characteristics of the included studies in the systematic review.

No.	Study ID (Ref.)	Country of Study	Period of Study	Source of Samples	No. of *S.* Typhi Isolates	No. of Resistant *S.* Typhi	Phenotypic Resistance (n)	Genotypic Resistance (n)
1	Abbasi & Ghaznavi-Rad, 2021 [12]	Iran	2015–2016	Patient/clinical	3	3	AMP (1), CHL (1), NAL (3), SXT (1), TET (2)	*gyrA* (3), *parC* (3), *int1* (2), *qac* (1), *qnrS1* (3), *qnrA* (3), *qnrB* (1), *sul1* (1)
2	Afzal et al., 2012 [13]	Pakistan	2011	Patient/clinical	30	14	CFM (4), CIP (3), CPD (5), CRO (1), NAL (9), RAD (14),	*gyrA* (9), *bla_TEM_* (14)
3	Afzal et al., 2013 [14]	Pakistan	2010–2011	Patient/clinical	80	80	AMK (12), AMP (45), ATM (12), CFM (10), CFP-SUL (2), CHL (26), CIP (10), CRO (9), GEN (5), NAL (19), RAD (31), STR (42), SXT (24), TET (23), TMP (23)	*bla_TEM_* (35), *catA1* (21), *dfrA7* (30), *strA* (21), *strB* (21), *sul1* (24), *sul2* (54), *tetB* (28)
4	Ahsan & Rahman, 2019 [15]	Bangladesh	2015–2016	Patient/clinical	33	33	AZM (31), CLI (33),	*ermB* (21), *int1* (29)
5	Akinyemi et al., 2011 [16]	Nigeria	NA	Environmental	3	3	AMP (3), AMX (1), CHL (2), GEN (2), STR (3), SXT (1), TET (3)	*stn* (3)
6	Akinyemi et al., 2017 [17]	Western Africa	2014–2015	Patient/clinical	13	12	FOX (12)	*ampC fox* (7)
7	AL-Fatlawy & AL-Hadrawi, 2020 [18]	Iraq	2018–2019	Patient/clinical	59	59	AMC (59), AMP (52), AMX (50), ATM (59), CAR (40), CHL (47), CLA (59), CLR (59), CPR (44), CTX (59), FOX (59), GEN (48), IPM (53), MEM (59), NIT (59), PEN (59), TET (59),	*int1* (43)
8	Aljanaby & Medhat, 2017 [19]	Iraq	2016–2017	Patient/clinical	39	39	AMK (5), AMP (24), AMX (18), CHL (20), CIP (2), CTX (1), GEN (4), STR (9), TET (17), TOB (9)	*ant (3″)-Ia* (1), *bla_TEM_* (6), *bla_SHV_* (5), *catA1* (24), *catA2* (8), *cmIA* (4), *floR* (29), *pse-1* (18), *tetA* (19), *tetB* (11)
9	Al-Mayahi & Jaber, 2020 [20]	Iraq	2018	Patient/clinical	56	56	AMC (31), AMK (21), AMP (56), ATM (18), AZM (51), CAZ (28), CFM (51), CHL (56), CIP (30), CPD (56), CRO (22), CTX (39), CXM (56), DOX (35), FEP (14), FOX (56), GEN (17), LEX (56), LVX (10), MIN (35), NAL (56), NET (8), NIT (56), NOR (16), OFX (10), PIP (30), SAM (50), SXT (18), TET (56), TIC (24), TIM (13), TMP (22), TOB (17), TZP (14)	*bla_TEM_* (40), *bla_CTX-M1_* (27), *bla_SHV_* (11)
10	Al-Muhanna et al., 2018 [21]	Iraq	NA	Patient/clinical	30	30	NA	*gyrA* (30), *gyrB* (25), *catA1* (19), *dfrA7* (18), *sul1* (12), *sul2* (6)
11	Baucheron et al., 2014 [22]	France	1997–2008	Patient/clinical	16	14	AMX (7), CHL (7), CIP (1), NAL (14), SPT (1), STR (8), SXT (8), TET (8), TMP (8)	*gyrA* (14), *parC* (1), *parE* (2)
12	Brown et al., 1996 [23]	India	1992–1994	Patient/clinical	15	12	NAL (12)	*gyrA* (12)
13	Chattaway et al., 2021 [24]	England	2016–2019	Patient/clinical	1034	956	AMC (308), AMX (308), CAZ (51), CHL (323), CIP (950), CRO (51), FOF (1), SXT (336), TET (28), TMP (328)	*gyrA* (913), *gyrB* (36), *parC* (150), *parE* (24), *bla_TEM_* (303), *bla_CTX-M15_* (49), *bla_SHV_* (1), *catA1* (323), *dfrA7*(317), *dfrA14* (12), *dfrA15* (8), *fosA-v3* (1), *qnrS1* (55), *strA* (301), *strB* (298), *sul1* (327), *sul2* (302)
14	Dahiya et al., 2014 [25]	India	2005–2010	Patient/clinical	22	17	CIP (6), NAL (17)	*gyrA* (17), *parC* (6)
15	Day et al., 2018 [26]	United Kingdom	2014–2016	Patient/clinical	332	292	AMP (77), CHL (79), CIP (36), STR (76), SXT (83), TET (6), TMP (82)	*gyrA* (275), *gyrB* (7), *parC* (45), *parE* (6), *bla_TEM_* (77), *catA1* (79), *dfrA1* (1), *dfrA7* (74), *dfrA14* (1), *dfrA15* (6), *qnrB* (1), *strA* (76), *strB* (76), *sul1* (81), *sul2* (76), *tetA* (6)
16	El-Tayeb et al., 2017 [27]	Saudi Arabia	NA	Patient/clinical and environmental	4	4	AMK (4), CEF (4), CXM (4), FOX (4), GEN (4), NIT (4), SXT (4), TOB (4)	*gyrA* (1), *parC* (1), *carb* (3), *dfrA1* (2), *floR* (4), *tetA* (1), *tetG* (4)
17	Eshaghi et al., 2020 [28]	Canada	2018–2019	Patient/clinical	10	10	AMP (10), CHL (10), CIP (10), CRO (10), STR (10), SXT (10)	*gyrA* (10), *aac (6′)-Iaa* (10), *aph (3″)-Ib* (10), *aph (6)-Id* (10), *bla_TEM_* (10), *bla_CTX-M15_* (10), *catA1* (10), *dfrA7* (10), *qnrS1* (10), *sul1* (10), *sul2* (10), *tetA* (1)
18	Gaind et al., 2006 [29]	India	2003–2004	Patient/clinical	8	6	CIP (3), NAL (6), SXT (4), TET (4)	*gyrA* (5), *parC* (5), *aadA1* (4), *dfrA15* (4), *int1* (4)
19	García et al., 2014 [30]	Peru	2008–2012	Patient/clinical	33	6	NAL (6)	*gyrA* (6)
20	García-Fernández et al., 2015 [31]	Italy	2011–2013	Patient/clinical	19	14	AMP (3), CHL (3), CIP (13), NAL (13), STR (4), SXT (2), TET (1), TMP (3)	*gyrA* (13), *gyrB* (5), *parC* (3), *parE* (1)
21	Gopal et al., 2016 [32]	India	2007–2009	Patient/clinical	133	132	AMP (4), CIP (28), NAL (132)	*gyrA* (125), *parC* (29)
22	Hassing et al., 2011 [33]	Netherland	2002–2008	Patient/clinical	11	8	AMP (2), CHL (2), CIP (1), NAL (8), TET (2), TMP (2)	*gyrA* (8)
23	Hassing et al., 2016 [34]	Netherland	2002–2008	Patient/clinical	11	8	CIP (8), NAL (8)	*gyrA* (8)
24	Jacob et al., 2021 [35]	India	2015–2018	Patient/clinical	5	5	AMP (5), CIP (5), CRO (5)	*gyrA* (5), *parC* (5), *aac (6′)-Iaa* (1), *bla_TEM_* (1), *bla_DHA_* (1), *bla_SHV_* (4), *qnrB* (5), *sul1* (1)
25	Lima et al., 2019 [36]	Bangladesh	NA	Patient/clinical	73	63	AMP (38), CHL (39), CIP (59), CRO (1), SXT (35)	*gyrA* (62), *gyrB* (4), *parC* (4), *parE* (6), *bla_TEM_* (57), *bla_CTX-M15_* (1), *catA1* (39), *dfrA7* (40), *qnrS1* (7), *sul1* (39), *sul2* (36)
26	Lv, Zhang & Song, 2019 [37]	China	2005–2017	Patient/clinical	140	95	AMP (74), NAL (95)	*gyrA* (87), *bla_TEM_* (74)
27	Matono et al., 2017 [38]	Japan	2001–2016	Patient/clinical	107	47	CIP (47)	*gyrA* (47), *parC* (37), *parE* (3)
28	Nüesch-Inderbinen et al., 2015 [39]	Switzerland	2002–2013	Patient/clinical	192	126	AMC (1), AMP (27), CEF (8), CHL (24), CIP (39), NAL (113), STR (25), SXT (35), TET (15), TMP (28)	*gyrA* (35), *parC* (9), *qnrS1* (2)
29	Okanda et al., 2018 [40]	Bangladesh	2015	Patient/clinical	18	18	AMP (7), CHL (7), NAL (18), SXT (7), TET (5)	*gyrA* (18), *parC* (1)
30	Oo et al., 2019 [41]	Myanmar	2015–2016	Patient/clinical	39	39	CIP (39)	*gyrA* (39), *gyrB* (1), *parC* (16)
31	Qian et al., 2020 [42]	China	2013–2017	Patient/clinical	164	94	AMC (3), AMP (16), CAZ (2), CIP (4), CRO (3), CTF (10), CTX (2), GEN (2), NAL (94), SXT (2), TET (5)	*gyrA* (68), *gyrB* (1), *parC* (1), *parE* (5), *qnrS1* (1), *qnrB* (1)
32	Saeed et al., 2020 [43]	Pakistan	2018	Patient/clinical	82	82	AMP (61), AMX (61), CFM (35), CHL (79), CIP (74), CRO (35), CTX (35), FEP (35), SXT (79), TZP (31)	*bla_TEM_* (42), *bla_CTX-M1_* (27), *bla_CTX-M15_* (21)
33	Shah et al., 2020 [44]	Pakistan	NA	Environmental	110	108	AMK (108), ATM (107), CIP (66), CRO (108), ETP (108), IPM (107), MEM (108), PEN (108), VAN (108)	*gyrA* (44)
34	Sharma et al., 2019 [45]	India	1993–2016	Patient/clinical	469	32	AZM (32)	*acrR* (1), *rpIV* (2)
35	Shirakawa et al., 2006 [46]	Nepal	2003	Patient/clinical	30	23	AMP (9), CHL (7), NAL (22), SXT (7), TET (5)	*gyrA* (22)
36	Smith, Govender & Keddy, 2010 [47]	South Africa	2003–2007	Patient/clinical	19	19	NAL (19)	*gyrA* (7), *parC* (7)
37	Song et al., 2010 [48]	Worldwide	1991–2006	Patient/clinical	292	223	NAL (223)	*gyrA* (213), *parE* (33)
38	Tanmoy et al., 2018 [49]	Bangladesh	1999–2013	Patient/clinical	536	474	AMP (263), CHL (250), CIP (467), CRO (1), SXT (233)	*gyrA* (458), *gyrB* (17), *parC* (17), *parE* (68), *bla_TEM_* (271), *bla_CTX-M1_* (1), *catA1* (256), *dfrA7* (257), *qnrS1* (255), *strA* (210), *strB* (210), *sul1* (257), *sul2* (265), *tetA* (51), *tetB* (46)
39	Veeraraghavan et al., 2016 [50]	India	2014	Patient/clinical	27	25	AMP (2), CIP (10), NAL (24), PEF (25), SXT (2)	*gyrA* (24), *parC* (14), *qnrB* (1)
40	Vlieghe et al., 2012 [51]	Cambodia	2007–2010	Patient/clinical	20	18	AZM (1), NAL (18),	*gyrA* (18)
41	Wu et al., 2010 [52]	China	2002–2007	Patient/clinical	25	13	AMP (1), NAL (13)	*gyrA* (13)
42	Yanagi et al., 2009 [53]	Indonesia	2006–2008	Patient/clinical	17	8	AMP (8), CHL (3), CIP (3), CRO (1), LVX (1), NAL (8), SXT (3), TET (1)	*gyrA* (8)

Numbers in parentheses, (n), indicate the number of isolates. AMC: amoxicillin-clavulanic acid, AMK: amikacin, AMP: ampicillin, AMX: amoxicillin, ATM: aztreonam, AZM: azithromycin, CAR: carbenicillin, CAZ: ceftazidime, CEF: cephalothin, CFM: cefixime, CFP-SUL: cefoperazone-sulbactam, CHL: chloramphenicol, CIP: ciprofloxacin, CLA: clavulanic acid, CLI: clindamycin, CLR: clarithromycin, CPD: cefpodoxime, CPR: cefpirome, CRO: ceftriaxone, CTF: ceftiofur, CTX: cefotaxime, CXM: cefuroxime, DOX: doxycycline, ETP: ertapenem, FEP: cefepime, FOF: fosfomycin, FOX: cefoxitin, GEN: gentamicin, IPM: imipinem, LEX: cephalexin, LVX: levofloxacin, MEM: meropenem, MIN: minocycline, NA: not applicable, NAL: nalidixic acid, NET: netilmicin, NIT: nitrofurantoin, NOR: norfloxacin, OFX: ofloxacin, PEF: pefloxacin, PEN: penicillin, PIP: piperacillin, RAD: cephradine, SAM: ampicillin-sulbactam, SPT: spectinomycin, STR: streptomycin, SXT: trimethoprim-sulfamethoxazole, TET: tetracycline, TIC: ticarcillin, TIM: ticarcillin-clavulanic acid, TMP: trimethoprim, TOB: tobramycin, TZP: piperacillin-tazobactam, VAN: vancomycin.

**Table 2:** Sub-group analysis of prevalence of resistant *S.* Typhi according to countries and regions.

Country or Region of Study	No. of Studies	Prevalence (%)	95% CI	*I* ^2^	Q	Heterogeneity Test	
						DF	*p*
Country							
Bangladesh	4	88.7	83.7–92.3	22.168	3.854	3	0.278
Cambodia	1	90.0	67.6–97.5	0.000	0.000	0	1.000
Canada	1	95.5	55.2–99.7	0.000	0.000	0	1.000
China	3	60.8	51.7–69.3	55.694	4.514	2	0.105
England	1	92.5	90.7–93.9	0.000	0.000	0	1.000
France	1	87.5	61.4–96.9	0.000	0.000	0	1.000
India	7	81.5	30.4–97.8	96.529	172.877	6	0.000
Indonesia	1	47.1	25.5–69.7	0.000	0.000	0	1.000
Iran	1	87.5	26.6–99.3	0.000	0.000	0	1.000
Iraq	4	98.9	95.7–99.7	0.000	0.147	3	0.986
Italy	1	73.7	50.2–88.6	0.000	0.000	0	1.000
Japan	1	43.9	34.8–53.4	0.000	0.000	0	1.000
Myanmar	1	98.8	82.9–99.9	0.000	0.000	0	1.000
Nepal	1	76.7	58.5–88.4	0.000	0.000	0	1.000
Netherland	2	72.7	51.1–87.2	0.000	0.000	1	1.000
Nigeria	2	91.0	65.3–98.2	0.000	0.086	1	0.769
Pakistan	4	94.8	59.5–99.6	93.686	47.513	3	0.000
Peru	1	18.2	8.4–35.0	0.000	0.000	0	1.000
Saudi Arabia	1	90.0	32.6–99.4	0.000	0.000	0	1.000
South Africa	1	97.5	70.2–99.8	0.000	0.000	0	1.000
Switzerland	1	65.6	58.6–72.0	0.000	0.000	0	1.000
United Kingdom	1	88.0	84.0–91.0	0.000	0.000	0	1.000
Worldwide	1	76.4	71.2–80.9	0.000	0.000	0	1.000
Region							
Africa	3	93.5	77.3–98.4	0.000	0.742	2	0.690
America	2	63.7	2.0–99.3	88.889	9.000	1	0.003
Asia	23	83.5	70.7–91.4	96.156	572.296	22	0.000
Europe	7	81.4	66.9–90.4	93.997	99.949	6	0.000
Middle East	6	97.7	93.0–99.3	0.000	3.761	5	0.584
Worldwide	1	76.4	71.2–80.9	0.000	0.000	0	1.000

**Table 3:** Sub-group analysis of prevalence of *gyrA* gene in *S.* Typhi according to countries and regions.

Country or Region of Study	No. of Studies	Prevalence (%)	95% CI	*I* ^2^	Q	Heterogeneity Test	
						DF	*p*
Country							
Bangladesh	3	96.8	94.9–98.0	0.000	0.574	2	0.751
Cambodia	1	97.4	69.0–99.8	0.000	0.000	0	1.000
Canada	1	95.5	55.2–99.7	0.000	0.000	0	1.000
China	3	86.8	64.3–96.0	83.974	12.480	2	0.002
England	1	95.5	94.0–96.6	0.000	0.000	0	1.000
France	1	96.7	63.4–99.8	0.000	0.000	0	1.000
India	6	94.4	90.1–96.9	0.000	1.790	5	0.877
Indonesia	1	94.4	49.5–99.7	0.000	0.000	0	1.000
Iran	1	87.5	26.6–99.3	0.000	0.000	0	1.000
Iraq	1	98.4	78.9–99.9	0.000	0.000	0	1.000
Italy	1	92.9	63.0–99.0	0.000	0.000	0	1.000
Japan	1	99.0	85.4–99.9	0.000	0.000	0	1.000
Myanmar	1	98.8	82.0–99.9	0.000	0.000	0	1.000
Nepal	1	95.7	74.8–99.4	0.000	0.000	0	1.000
Netherland	2	94.4	69.3–99.2	0.000	0.000	1	1.000
Pakistan	2	49.1	28.2–70.4	62.276	2.651	1	0.103
Peru	1	92.9	42.3–99.6	0.000	0.000	0	1.000
Saudi Arabia	1	25.0	3.4–76.2	0.000	0.000	0	1.000
South Africa	1	36.8	18.7–59.7	0.000	0.000	0	1.000
Switzerland	1	27.8	20.7–36.2	0.000	0.000	0	1.000
United Kingdom	1	94.2	90.8–96.4	0.000	0.000	0	1.000
Worldwide	1	95.5	91.9–97.6	0.000	0.000	0	1.000
Region							
Africa	1	36.8	18.7–59.7	0.000	0.000	0	1.000
America	2	94.3	68.7–99.2	0.000	0.054	1	0.816
Asia	19	93.2	85.2–97.0	91.108	202.421	18	0.000
Europe	7	90.5	61.2–98.3	97.817	274.892	6	0.000
Middle East	3	82.7	17.0–99.1	76.116	8.374	2	0.015
Worldwide	1	95.5	91.9–97.6	0.000	0.000	0	1.000

**Table 4:** Sub-group analysis of prevalence *gyrB* gene in *S.* Typhi according to countries and regions.

Country or Region of Study	No. of Studies	Prevalence (%)	95% CI	*I* ^2^	Q	Heterogeneity Test	
						DF	*p*
Country							
Bangladesh	2	4.1	2.5–6.4	8.969	1.099	1	0.295
China	1	1.1	0.1–7.2	0.000	0.000	0	1.000
England	1	3.8	2.7–5.2	0.000	0.000	0	1.000
Iraq	1	83.3	65.7–92.9	0.000	0.000	0	1.000
Italy	1	35.7	15.7–62.4	0.000	0.000	0	1.000
Myanmar	1	2.6	0.4–16.1	0.000	0.000	0	1.000
United Kingdom	1	2.4	1.1–4.9	0.000	0.000	0	1.000
Region							
Asia	4	3.7	2.5–5.5	0.000	2.969	3	0.396
Europe	3	6.9	1.9–22.5	91.477	23.467	2	0.000
Middle East	1	83.3	65.7–92.9	0.000	0.000	0	1.000

**Table 5:** Sub-group analysis of prevalence *parC* gene in *S.* Typhi according to countries and regions.

Country or Region of Study	No. of Studies	Prevalence (%)	95% CI	*I* ^2^	Q	Heterogeneity Test	
						DF	*p*
Country							
Bangladesh	3	4.1	2.7–6.1	0.000	1.207	2	0.547
China	1	1.1	0.1–7.2	0.000	0.000	0	1.000
England	1	15.7	13.5–18.1	0.000	0.000	0	1.000
France	1	7.1	1.0–37.0	0.000	0.000	0	1.000
India	5	50.0	25.6–74.4	81.214	21.293	4	0.000
Iran	1	87.5	26.6–99.3	0.000	0.000	0	1.000
Italy	1	21.4	7.1–49.4	0.000	0.000	0	1.000
Japan	1	78.7	64.8–88.2	0.000	0.000	0	1.000
Myanmar	1	41.0	26.9–56.8	0.000	0.000	0	1.000
Saudi Arabia	1	25.0	3.4–76.2	0.000	0.000	0	1.000
Region							
Asia	11	27.1	11.3–52.0	94.328	176.293	10	0.000
Europe	5	14.0	10.8–18.0	44.721	7.236	4	0.124
Middle East	2	56.6	6.3–96.2	60.956	2.561	1	0.110
Africa	1	36.8	18.7–59.7	0.000	0.000	0	1.000
South Africa	1	36.8	18.7–59.7	0.000	0.000	0	1.000
Switzerland	1	7.1	3.8–13.2	0.000	0.000	0	1.000
United Kingdom	1	15.4	11.7–20.0	0.000	0.000	0	1.000

**Table 6:** Sub-group analysis of prevalence *parE* gene in *S.* Typhi according to regions.

Country or Region of Study	No. of Studies	Prevalence (%)	95% CI	*I* ^2^	Q	Heterogeneity Test	
						DF	*p*
Country							
Bangladesh	2	13.7	10.6–17.5	6.644	1.071	1	0.301
China	1	5.3	2.2–12.1	0.000	0.000	0	1.000
England	1	2.5	1.7–3.7	0.000	0.000	0	1.000
France	1	14.3	3.6–42.7	0.000	0.000	0	1.000
Italy	1	7.1	1.0–37.0	0.000	0.000	0	1.000
Japan	1	6.4	2.1–18.0	0.000	0.000	0	1.000
United Kingdom	1	2.1	0.9–4.5	0.000	0.000	0	1.000
Worldwide	1	14.8	10.7–20.1	0.000	0.000	0	1.000
Region							
Asia	4	9.4	5.5–15.6	60.644	7.623	3	0.054
Europe	4	3.6	1.7–7.2	57.466	7.053	3	0.070
Worldwide	1	14.8	10.7–20.1	0.000	0.000	0	1.000

**Table 7:** Sub-group analysis of prevalence *bla_TEM_* gene in *S.* Typhi according to countries and regions.

Country or Region of Study	No. of Studies	Prevalence (%)	95% CI	*I* ^2^	Q	Heterogeneity Test	
						DF	*p*
Country							
Bangladesh	2	77.3	33.3–95.9	94.993	19.971	1	0.000
Canada	1	95.5	55.2–99.7	0.000	0.000	0	1.000
China	1	77.9	68.5–85.1	0.000	0.000	0	1.000
England	1	31.7	28.8–34.7	0.000	0.000	0	1.000
India	1	20.0	2.7–69.1	0.000	0.000	0	1.000
Iraq	2	40.8	5.0–90.0	95.859	24.150	1	0.000
Pakistan	3	52.4	35.2–68.9	69.880	6.640	2	0.036
United Kingdom	1	26.4	21.6–31.7	0.000	0.000	0	1.000
Region							
Asia	7	65.3	50.9–77.3	87.849	49.379	6	0.000
Europe	2	29.5	24.6–34.9	66.500	2. 985	1	0.084
Middle East	2	40.8	5.0–90.0	95.859	24.150	1	0.000
America	1	95.5	55.2–99.7	0.000	0.000	0	1.000

## Data Availability

The datasets used and/or analyzed during the current study are included in the manuscript.

## Appendix A

**Table A1:** Quality of included studies by JBI critical appraisal checklist for studies reporting prevalence data.

No.	Author	Checklist									Score
		1	2	3	4	5	6	7	8	9	
1	Abbasi & Ghaznavi-Rad, 2021 [12]	YES	YES	YES	YES	YES	YES	NO	NO	YES	7
2	Afzal et al., 2012 [13]	YES	YES	YES	YES	NO	YES	YES	YES	NO	7
3	Afzal et al., 2013 [14]	YES	YES	YES	YES	NO	YES	YES	NO	YES	7
4	Ahsan & Rahman, 2019 [15]	YES	YES	YES	YES	NO	YES	YES	NO	YES	7
5	Akinyemi et al., 2011 [16]	YES	YES	YES	YES	YES	YES	YES	YES	YES	9
6	Akinyemi et al., 2017 [17]	YES	YES	YES	YES	YES	YES	YES	YES	YES	9
7	Al-Fatlawy & Al-Hadrawi, 2020 [18]	YES	YES	YES	NO	NO	YES	YES	YES	YES	7
8	Aljanaby & Medhat, 2017 [19]	YES	YES	YES	YES	YES	YES	YES	YES	YES	9
9	Al-Mayahi & Jaber, 2020 [20]	YES	YES	YES	YES	YES	YES	YES	YES	YES	9
10	Al-Muhanna et al., 2018 [21]	YES	YES	YES	YES	NO	YES	YES	NO	YES	7
11	Baucheron et al., 2014 [22]	YES	NO	YES	YES	YES	YES	YES	NO	YES	7
12	Brown et al., 1996 [23]	NO	NO	YES	YES	YES	YES	YES	YES	YES	7
13	Chattaway et al., 2021 [24]	YES	YES	YES	YES	NO	YES	YES	YES	YES	8
14	Dahiya et al., 2014 [25]	YES	YES	YES	NO	YES	YES	YES	YES	YES	8
15	Day et al., 2018 [26]	YES	YES	YES	YES	YES	YES	YES	YES	NO	8
16	El-Tayeb et al., 2017 [27]	YES	YES	YES	YES	YES	YES	YES	YES	YES	9
17	Eshaghi et al., 2020 [28]	YES	YES	YES	YES	NO	YES	YES	NO	YES	7
18	Gaind et al., 2006 [29]	YES	YES	YES	NO	YES	YES	YES	NO	YES	7
19	García et al., 2014 [30]	YES	NO	YES	YES	YES	YES	YES	NO	YES	7
20	García-Fernández et al., 2015 [31]	YES	YES	YES	YES	NO	YES	YES	YES	YES	8
21	Gopal et al., 2016 [32]	YES	YES	YES	NO	NO	YES	YES	YES	YES	7
22	Hassing et al., 2011 [33]	YES	YES	YES	YES	NO	YES	YES	YES	YES	8
23	Hassing et al., 2016 [34]	YES	YES	YES	YES	NO	YES	YES	YES	NO	7
24	Jacob et al., 2021 [35]	YES	YES	YES	YES	YES	YES	NO	YES	YES	8
25	Lima et al., 2019 [36]	YES	YES	YES	YES	YES	YES	YES	YES	YES	9
26	Lv, Zhang & Song, 2019 [37]	YES	YES	YES	YES	NO	YES	YES	YES	YES	8
27	Matono et al., 2017 [38]	YES	YES	YES	YES	YES	YES	YES	YES	YES	9
28	Nüesch-Inderbinen et al., 2015 [39]	YES	YES	YES	YES	NO	YES	YES	NO	YES	7
29	Okanda et al., 2018 [40]	YES	YES	YES	YES	NO	YES	YES	YES	YES	8
30	Oo et al., 2019 [41]	YES	YES	YES	YES	NO	YES	YES	NO	YES	7
31	Qian et al., 2020 [42]	YES	YES	YES	YES	NO	YES	YES	YES	YES	8
32	Saeed et al., 2020 [43]	YES	YES	YES	YES	NO	YES	YES	NO	YES	7
33	Shah et al., 2020 [44]	YES	YES	YES	YES	NO	YES	YES	NO	YES	7
34	Sharma et al., 2019 [45]	YES	YES	YES	YES	NO	YES	YES	YES	NO	7
35	Shirakawa et al., 2006 [46]	YES	YES	YES	YES	NO	YES	YES	YES	YES	8
36	Smith, Govender & Keddy, 2010 [47]	YES	YES	YES	YES	NO	YES	YES	NO	YES	7
37	Song et al., 2010 [48]	YES	YES	YES	YES	YES	YES	YES	YES	YES	9
38	Tanmoy et al., 2018 [49]	YES	YES	YES	YES	YES	YES	YES	YES	YES	9
39	Veeraraghavan et al., 2016 [50]	YES	YES	YES	YES	NO	YES	YES	YES	YES	8
40	Vlieghe et al., 2012 [51]	YES	YES	YES	YES	YES	YES	YES	YES	YES	9
41	Wu et al., 2010 [52]	YES	YES	YES	NO	NO	YES	YES	YES	YES	7
42	Yanagi et al., 2009 [53]	YES	YES	YES	YES	YES	YES	YES	NO	YES	8

-Checklist questions: (1). Was the sample frame appropriate to address the target population?; (2). Were study participants sampled in appropriate way?; (3). Was the sample size adequate?; (4). Were the study subjects and the setting described in?; (5). Was a sample size justification, power description, or variance and effect estimates provided?; (6). Were valid methods used for the identification of the condition?; (7). Was the condition measured in a standard, reliable way for all participants?; (8). Was there appropriate statistical analysis?; (9). Was the response rate adequate, and if not, was the low response rate managed appropriately? -Score: ‘1’ for ‘yes’, ‘0’ for ‘no’; score ‘7’ to ‘9’ were of sufficient quality.

## Appendix B

**Table A2:** Distribution of mutations in *gyrA*, *gyrB*, *parC* and *parE* genes.

No.	Study ID (Ref.)	Mutations (n)			
		*gyrA* Gene	*gyrB* Gene	*parC* Gene	*parE* Gene
1	Abbasi & Ghaznavi-Rad, 2021 [12]	S83L (3)	NA	S80I (3)	NA
2	Afzal et al., 2012 [13]	S83F (9)	NA	NA	NA
3	Baucheron et al., 2014 [22]	S83F (10), S83Y (2), D87G (1), D87N (2)	NA	S80I (1)	D420N (2)
4	Brown et al., 1996 [23]	S83F (10), D87Y (2)	NA	NA	NA
5	Chattaway et al., 2021 [24]	S83F (768), D87V (4), S83Y (119), D87G (8), D87N (131), E133G (6), A119E (1), V85A (1), D87X (1)	S464Y (9), S464F (22)	S80I (127), E84G (11), E84K (6), G78D (1), D79G (4), Y74X (1), P98X (1)	E460D (1), S458A (23)
6	Dahiya et al., 2014 [25]	S83F (13), S83Y (3), D87G (1), D87N (6)	NA	S80I (6)	NA
7	Day et al., 2018 [26]	S83F (205), S83Y (50), D87Y (4), D87G (5), D87N (42)	S464F (7)	S80I (33), E84G (2), E84K (3), G78D (2), D79G (5)	E460K (3), S458A (2), L502F (1)
8	Eshaghi et al., 2020 [28]	S83F (10)	NA	NA	NA
9	Gaind et al., 2006 [29]	S83F (5), D87N (3)	NA	S80I (4), D69E (1)	NA
10	García et al., 2014 [30]	S83F (3), D87N (3)	NA	NA	NA
11	García-Fernández et al., 2015 [31]	S83F (10), S83Y (1), D87N (3), D82N (1)	G435A (1), G435E (3), G435V (1)	S80I (2), T57S (1)	S493F (1)
12	Gopal et al., 2016 [32]	S83F (125)	NA	W106G (29)	NA
13	Hassing et al., 2011 [33]	S83F (6), S83Y (2)	NA	NA	NA
14	Hassing et al., 2016 [34]	S83F (6), S83Y (2)	NA	NA	NA
15	Jacob et al., 2021 [35]	S83F (5), D87N (4)	NA	S80I (4), E84K (1)	NA
16	Lima et al., 2019 [36]	S83F (27), S83Y (29), D87G (2), D87N (3), D538N (51), N529S (3)	S464F (4)	S80I (1), S80E (1), E84K (2)	A364V (5), L416F (1), S339L (1)
17	Lv, Zhang & Song, 2019 [37]	S83F (62), D87Y (25)	NA	NA	NA
18	Matono et al., 2017 [38]	S83F (47), D87N (33)	NA	S80I (33), E84G (4)	D420N (3)
19	Nüesch-Inderbinen et al., 2015 [39]	S83F (27), S83Y (8), D87N (7)	NA	S80I (7), E84G (1), E84K (1)	NA
20	Okanda et al., 2018 [40]	S83F (12), S83Y (6)	NA	D69A (1)	NA
21	Oo et al., 2019 [41]	S83F (39), D87N (16)	G342E (1)	S80I (16)	NA
22	Qian et al., 2020 [42]	S83F (39), S83Y (5), D87G (3), D87N (18), E133G (59), S87G (1), D79G (3)	S426G (1)	E84K (1)	I444S (5), Y434S (1)
23	Shirakawa et al., 2006 [46]	S83F (22)	NA	NA	NA
24	Smith, Govender & Keddy, 2010 [47]	S83F (1), D82G (3), A119S (1), S83A (2), S83M (1), D87C (1), A119G (1), G81S (1)	NA	S80I (1), S80F (1), S80K (1), T57A (1), T57G (1), T57S (1), S80R (3)	NA
25	Song et al., 2010 [48]	S83F (176), S83Y (27), D87G (10)	NA	NA	D420N (33)
26	Tanmoy et al., 2018 [49]	S83F (290), D87Y (2), S83Y (124), D87G (10), D87N (29), A119E (1), D538N (346), N529S (6)	S464Y (7), S464F (7)	S80I (1), E84G (1), E84K (10), D69A (2), S80R (2), T620M (1)	E460K (1), L502F (1), A364V (53), L416F (2), T447A (9), S339L (2), A365S (2)
27	Veeraraghavan et al., 2016 [50]	S83F (17), S83Y (5), D87Y (2), D87N (8)	NA	S80I (8), E84G (2), E84K (2), G72S (2)	NA
28	Vlieghe et al., 2012 [51]	S83F (18), E133G (18)	NA	NA	NA
29	Wu et al., 2010 [52]	S83F (9), D87G (1), D87N (3)	NA	NA	NA
30	Yanagi et al., 2009 [53]	D87Y (8)	NA	NA	NA

Numbers in parentheses, ( ), indicate the number of isolates. A: Alanine, C: cysteine, D: aspartic acid, E: glutamic acid, F: phenylalanine, G: glycine, I: isoleucine, K: lysine, L: leucine, M: methionine, N: asparagine, NA: not applicable, P: proline, R: arginine, S: serine, T: threonine, V: valine, W: tryptophan, X: any amino acid, Y: tyrosine.
